# Genome‐wide and gene‐specific DNA methylation across developmental stages in *Pogonomyrmex californicus*: A socially polymorphic ant

**DOI:** 10.1111/imb.70021

**Published:** 2025-12-20

**Authors:** Tania Chavarria‐Pizarro, Mohammed Errbii, Janina Rinke, Lukas Schrader, Jürgen Gadau

**Affiliations:** ^1^ Institute for Evolution and Biodiversity University of Münster Münster Germany; ^2^ Department of Biology University of Cologne Cologne Germany

**Keywords:** epigenetics, phenotypic plasticity, social insects

## Abstract

DNA methylation has been proposed as an epigenetic driver of phenotypic plasticity in social insects, yet experimental evidence remains limited. Even less is known about the role of epigenetic mechanisms underlying behavioural and social polymorphism. We quantified CpG methylation for the socially polymorphic harvester ant *Pogonomyrmex californicus* across larvae, pupae, workers and queens using Oxford Nanopore Technologies (ONT) sequencing. These results were compared against the current gold standard whole‐genome bisulfite sequencing (WGBS). Methylation sites were highly correlated between WGBS and ONT, validating the use of ONT for high‐throughput epigenomic profiling. Genome‐wide methylation was low (~3%), consistent with findings in other (Hymenoptera: Formicidae), and highly clustered within gene bodies, especially exons, while introns, intergenic DNA, promoters and transposable elements were hypo‐methylated. Gene body methylation (GBM) correlated positively with gene expression in queens, corroborating previous reports for other insects, suggesting a conserved regulatory role for DNA methylation in insects. A comparison between developmental stages revealed significant stage‐specific differences in GBM frequencies. Workers and queens, although from different populations, shared a substantial core of methylated loci enriched for olfactory‐receptor activity and biosynthetic pathways, processes that are central to caste‐specific behaviour and physiology. These shared methylation signatures, coupled with stage‐dependent variability, highlight DNA methylation as a possible factor in developmental stages and caste differentiation. In the future, it is essential to disentangle the effects of caste and variation between populations. Our study establishes *P. californicus* as a powerful model for dissecting how epigenetic modifications interface with gene expression to generate developmental and complex social organization, which is largely unexplored.

## Graphical abstract

- Comparison between developmental stages (larvae, pupae, worker) in *Pogonomyrmex californicus* revealed significant stage‐specific differences in Gene Body Methylated frequencies.
- Methylation sites were highly correlated between WGBS and ONT in *P. californicus* Genome‐wide methylation was low (~3%) and highly clustered within gene bodies (GBM), especially exons, while introns, intergenic DNA, promoters, transposable elements were hypo‐methylated.
- Gene body methylated (GBM) correlated positively with gene expression in queens, corroborating previous reports for other insects, suggesting a conserved regulatory role for DNA methylation in insects.

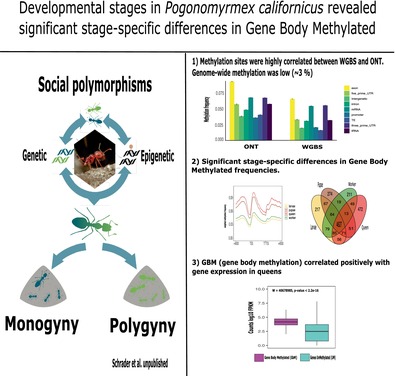

## INTRODUCTION

Epigenetics is the study of phenotypic changes in organisms generated by alterations of gene expression rather than changes to the DNA sequence (Bird, 2007; Deans & Maggert, 2015; Holliday & Pugh, 1975; Jablonka & Lamb, 2002; Richards, 2006). Epigenetic regulation includes DNA methylation, histone modification, chromatin remodelling, and ncRNA (Berger, 2007; Grant‐Downton & Dickinson, 2006). The ensemble of all epigenetic changes affecting the genome, also known as the epigenome, has been shown to have heritable components (Kungulovski & Jeltsch, 2016; Murrell et al., 2005).

Among epigenetic mechanisms, DNA methylation is arguably the most extensively studied mechanism (Duncan et al., 2014). In particular, the addition of a methyl group to the fifth cytosine's carbon (5mC) is the most common and well‐studied form of DNA methylation, and is found across a wide range of organisms including bacteria, plants, fungi, invertebrates and vertebrates (Duncan et al., 2014; Kungulovski & Jeltsch, 2016; Suzuki & Bird, 2008), where it has also been shown to regulate gene expression. However, its functions, biological characteristics, and genomic distribution are unique for each taxonomic lineage and vary considerably within each lineage (Colot & Rossignol, 1999). For instance, studies in vertebrates demonstrated that methylation of cytosines within a CpG context (5mCpG) reduced transcription of the methylated genes by blocking the binding of transcription factors to the DNA (Lyko, 2018). DNA methylation as control of the transposable elements (TEs) activity known as epigenetic silencing has been proposed as an important process to repress the potentially detrimental effects of TEs expression (Bestor, 1990). DNA methylation especially represses TEs in many higher eukaryotes (e.g., Deniz et al., 2019).

The role of DNA methylation in insects remains uncertain, varying widely across species and lineages. Several groups—including Diptera, some Lepidoptera, Coleoptera and Hymenoptera—have lost one or both DNA methyltransferase genes (*DNMT1* or *DNMT3*), showing little to no detectable methylation (Bewick et al., 2017). Such losses likely reflect lineage‐specific adaptations rather than a simple loss of epigenetic regulation, potentially driven by selective pressures for genome stability or the evolution of alternative mechanisms for gene regulation (Regev et al., 1998).

In social Hymenoptera, DNA methylation has been linked to caste differentiation through differential gene expression (Herb et al., 2012; Kucharski et al., 2008; Yan et al., 2015), although evidence remains mixed (Cardoso‐Junior et al., 2021; Lyko & Maleszka, 2011; Morandin et al., 2019; Oldroyd & Yagound, 2021; Patalano et al., 2015). Recent studies reveal Hymenoptera‐specific innovations in *DNMT3*, including duplication of the PWWP domain that binds H3K27me2/3 histone marks and the presence of non‐catalytic isoforms acting as auxiliary factors (Del Castillo Falconi et al., 2022; Duymich et al., 2016; Kucharski et al., 2023). These features suggest that DNMT proteins may have evolved broader regulatory functions—such as chromatin organization, transcriptional control, or developmental signalling—beyond their catalytic role in DNA methylation. Consistent with this idea, *DNMT1* and *DNMT3* show elevated expression in reproductive tissues of *Apis mellifera*, supporting additional roles in germline maintenance and early development (Lyko et al., 2010).

Phenotypic plasticity in eusocial insects is expressed not only through caste differentiation but also through pronounced intraspecific variation in the social organization of colonies. Colonies can range from strictly monogynous—headed by a single reproductive queen—to highly polygynous, harbouring several reproductive females (Boomsma et al., 2014; Keller, 1993; Saltz et al., 2016). Yet, comparative epigenomic studies that harness such social polymorphism to probe DNA‐methylation‐mediated social plasticity are missing.

The California harvester ant *Pogonomyrmex californicus* presents a powerful system for investigating the epigenetic basis of phenotypic plasticity and, in particular, social plasticity. Populations of this species display striking social polymorphism: colonies are either monogynous, headed by a single reproductive queen, or polygynous, comprising multiple—often unrelated—queens (Overson et al., 2016). These divergent social forms arise during colony founding. A newly mated queen may initiate a nest independently (haplometrosis), leading to monogyny, or multiple queens may cooperate to establish a nest (pleometrosis), resulting in primary polygyny (Johnson, 2004; Overson et al., 2016).

Because both social strategies occur within a single species, *P. californicus* provides an ideal system to explore whether DNA methylation contributes to both developmental and behavioural plasticity associated with alternative social organization—a largely unexplored topic of eusociality at the epigenomic level. Recent work by Errbi et al. (2024) further underscores *P. californicus*'s potential: their genomic analyses uncovered an 8 Mb non‐recombining region linked to the two social forms, analogous to supergene in other ants (Purcell et al., 2014; Wang et al., 2013). Intriguingly, this supergene contained DNMT3. Additionally, they also identified signals of selection in pleometrotic populations outside this region, including a highly differentiated genomic segment harbouring non‐synonymous substitutions in the DNA methyltransferase genes DNMT1. These findings highlight *P. californicus* as an emerging model for studying epigenetic regulation of phenotypic plasticity—not only in caste differentiation, but also in the emergence and maintenance of complex social behaviours.

Several techniques have been developed to detect epigenetic marks in natural populations, particularly DNA methylation (Husby, 2022). Whole Genome Bisulfite Sequencing (WGBS) is widely used, converting unmethylated cytosines into uracils via sodium bisulfite while leaving methylated cytosines unchanged. Oxford Nanopore Technologies (ONT) sequencing is gaining popularity as it avoids chemical treatment. Both methods are effective but have limitations. WGBS can degrade DNA and reduce coverage in GC‐rich regions due to bisulfite‐induced depyrimidination (Feng et al., 2020; Guanzon et al., 2024). ONT, while preserving DNA integrity, may show methylation bias from sequence context or low‐read coverage (Guanzon et al., 2024). Methylation accuracy also varies with the caller used, though biases diminish at coverage ≥10× (Guanzon et al., 2024; Yueng et al., 2021). Despite these caveats, ONT and WGBS show a strong correlation, especially when methylation is averaged across large genomic windows (1–100 kb) reflecting gene sizes (Perez et al., 2023). Notably, Faulk (2023) demonstrated that even low‐coverage ONT ‘genome skimming’ reliably estimates global methylation, with a 97% mapping rate and robust detection of transposon methylation due to ONT's long‐read capacity.

In this study, we present the first epigenetic survey of the harvester ant *P. californicus*, combining WGBS and ONT sequencing to profile genome‐wide DNA methylation. Our results demonstrated that *P. californicus* is a promising model to study the epigenetic mechanisms driving phenotypic plasticity. We show a high correlation between ONT sequencing and WGBS, using identical DNA pools (pupae) and assessing methylation patterns along functionally different genome regions, such as in TE‐rich regions (TEs islands), to shed light on the function and role of DNA methylation in insects. We further examined DNA methylation across three developmental stages (larva, pupa, adult) and two female castes (queens and workers), taking a crucial first step toward uncovering how epigenetic variation may shape phenotypic, behavioural plasticity, and social polymorphism in *P. californicus*. Finally, we found significant correlations between DNA methylation and gene expression in queens, providing insight into its potential regulatory role.

## METHODS

### Specimen collection and samples

For the study of developmental stage‐specific DNA methylation we used *P. californicus* (larva, pupae and workers) from queenright colonies kept at the Institute for Evolution and Biodiversity in Münster. Note, since these colonies do not produce sexual individuals, namely, males or virgin queens, we were sure that we investigated worker larvae and pupae. Those laboratory colonies were established from founding queens of *P. californicus* collected in 2018 and 2019 at Pine Valley (32.822 N, −116.528 W; pleometrotic population, P‐population) and Lake Henshaw resort (33.232 N, −116.760 W; haplometrotic population, H‐population), California, USA.

Additionally, we used founding queens, collected directly after their nuptial flight from a third population (Salt River, Phoenix, Arizona 2023; 33.55034 N, −111.64453 W). Overall, we prepared ONT libraries for ten queens and two DNA pools of 4 workers, two DNA pools of 4 pupae, and two DNA pools of 4 larvae each (Table 1). Each DNA pool was generated from individuals of the same colony. Finally, we used the same two pupal DNA pools for ONT libraries to generate two WGBS libraries, which allowed us to directly compare the results of ONT sequencing and WGBS.

**TABLE 1:** Nanopore ONT and WGBS sequencing—methylation data per stage and caste.

Stage	Method	*N*	Total called CpG sites	Methylated CpG sites	Average DNA methylation all CpG sites	Average DNA methylation frequency on genes (TSS–TTS)	No. methylated genes	No. un‐methylated genes
Larvae	ONT	2 pools (5 larvae each)	17,797,482	442,278	0.029	0.035	5024	10,875
Pupae	ONT	2 pools (4 small pupae)	533,737,549	11,865,786	0.031	0.080	5186	10,713
Pupae^a^	WGBS	2 pools (4 small pupae)	181,441,847	4,852,030	0.030	0.035	4760	9449
Worker	ONT	2 pools (4 each)	561,154,280	13,580,315	0.038	0.055	5320	9493
Queens	ONT	10	571,381,680	14,316,233	0.039	0.10	5520	10,639

*Note*: Average methylation was calculated as the average CG gene methylation across all genes. We calculated average DNA methylation for each stage and caste separately, as methylation levels differ between these categories. A gene was considered methylated if it had a significantly higher proportion of methylated cytosines than the genome‐wide methylation average at each stage or caste. This was performed using a Binomial test and *p*‐values were corrected for multiple tests.

Abbreviations: ONT, Oxford Nanopore Technologies; TSS, transcription start site; TTS, transcription end site; WGBS, whole‐genome bisulfite sequencing.

^a^ Same sample as pupae ONT.

### DNA isolation and sequencing

DNA was extracted from whole bodies of frozen or ethanol‐preserved individuals of *P. californicus* with the Qiagen DNeasy Blood and Tissue kit. A Qubit BR assay was used to assess DNA quantity, followed by 1% agarose gel electrophoresis to confirm the presence of high molecular weight DNA (>10 kb). We prepared DNA libraries for sequencing using the Oxford Nanopore Technologies (ONT) MinION platform according to the user manual. Following the manufacturer guidelines, we used PCR‐free Ligation Sequencing Kit (PCR‐free ONT Ligation Sequencing Kit with the Native Barcoding Expansion Kit [SQK‐LSK109 and EXPNBD103], and transposase‐based ONT Rapid Sequencing Kit [SQK‐RAD004]). Sequencing was done on FLO‐MIN 106D R9.4 flow cells, following the manufacturer's 1D native barcoding gDNA protocol. Each library was run on a separate FLO‐MIN 106D R9.4 flow cell. Basecalling fast 5 and demultiplexing were performed with ont‐guppy/6.4.8‐CUDA‐11.7.0 using the appropriate base‐calling model (dna_r9.4.1_450bps_hac.cfg) and default parameters (more details in https://github.com/TaniaChP79/P.cal-methylome) (Chavarria‐Pizarro et al., 2025a).

### Genome assemblies and annotations

We used the newest genome assembly and annotation for *P. californicus* (Pcal.3.1) (Errbii et al., 2021) based on combined ONT long‐read sequencing. The annotation included 15,899 protein‐coding genes and had a TE proportion of 22.79% (Errbii et al., 2021).

### CpG methylation calling

The Nanopolish v0.13.3 (Simpson et al., 2017) pipeline was used with default parameters to detect CpG methylation in ONT data. Nanopolish is computationally efficient and has previously been used in methylation studies using ONT sequencing data (Liu et al., 2021; Yueng et al., 2021). Briefly, the Nanopolish software uses a pre‐trained hidden Markov model to assign methylation log‐likelihood ratios (LLRs) to all CpGs within a 10 bp window. First, Nanopolish indexes the nanopore reads and then maps these onto the reference genome of *P. californicus* (Pcal 3.1) using minimap 2 v. 2.14 (Li, 2018). After alignment, the Nanopolish software analyses short CpG motif (11–34 bp) k‐mer sequences and separates 5‐methylcytosine from unmethylated cytosines based on signal disruptions in the raw ONT FAST5 sequence data. After that, the program calculates log‐likelihood ratios for base modifications of each read, where positive values indicate support for modification (methylation). We used the helper script *calculate_methylation_frequency.py* (Simpson et al., 2017) to convert the Nanopolish output into methylation frequency by genomic coordinates. The methylation frequency in Nanopolish is calculated as the proportion of reads that show evidence of methylation at a particular site relative to all reads covering that site. This provides a measure of how often a cytosine is methylated in the sampled population of molecules (Simpson et al., 2017).

The methylation calls were then subject to filtering using a minimum read count of 10 reads per CpG motif. We excluded genome sequences with methylation calls of less than 10% of CpGs in the sequence, as suggested by Perez et al. (2023). Additionally, a bed file with all CpGs for the genome was generated and mapped to the respective methylation annotations (call coverage and frequency) with the *bedtools map* function (Quinlan & Hall, 2010), both for the transcriptome annotation and the TE annotation (Errbii et al., 2021).

### Whole genome bisulfite sequencing

The WGBS sequencing libraries were prepared by Novogene (Munich, Germany). In short, paired‐end 150 bp bisulfite libraries were sequenced on the Illumina NovaSeq 6000 platform to a total of 35 million paired‐end reads (nuclear coverage >20×). Reads were trimmed with trimmomatic v0.30 (Bolger et al., 2014) (parameters: leading = 10, trailing = 10, minlen = 50) and processed with bismark v0.22.3 (Krueger & Andrews, 2011) to compute per‐base‐pair methylation frequencies. For WGBS data, we calculated methylation sites of pupae using the R package *methylKit v1.15.3* and R version 4.5.1 (Akalin et al., 2012). The percentage of methylated cytosines was calculated at a given site from the methylation ratios created by the software BSSeeker2, and complementary CpG dinucleotides were merged.

### Comparison between ONT and WGBS

We compared DNA methylation calls derived from ONT sequencing (sequencing depth >40× coverage) with whole‐genome bisulfite sequencing (WGBS, sequencing depth >20× coverage) from the same extracted DNA from pooled pupae (*n* = 2) that were used for the ONT sequencing. CpG motifs uniquely called either with Bismark or Nanopolish were further extracted using *bedtools subtract* (Quinlan & Hall, 2010). The per‐CpG motif methylation rates detected by ONT and WGBS were correlated using a Pearson correlation.

### TE annotation and inference of TE islands

TEs were annotated with RepeatMasker 4.1.7, using the newest assembly (Pcal 3.1). We created bed files of the TE and exon content across predefined genomic windows to infer TE islands. TE distribution was plotted for 50, 200 and 500 kb genomic windows using *bedtools makewindows*, *sortBed*, and *bedmap* from the bedtools package (Figure S1). TE islands were defined for the 16 chromosomes across 50 kb windows using a custom script in R, using changepoint modelling to call TE islands with the packages *EnvCpt*, *Rbeast*, *gdata*, *zoo* and *cowplot*. Parameters were set with a cutoff of 0.2 for the environment mean drop to call TE islands and a penalty of 40 for the environment mean model. Furthermore, we compared the average methylation frequency of TE islands with the rest of the genome using a Wilcoxon rank sum test. In addition, we compared the average methylation frequency of TEs on intragenetic areas, introns and a region closer to the gene (window of 2 kb upstream the gene region) to see if TE methylation changes along the genome. All visualizations and analyses were done in R.

### Inferences of gene body methylation

To exclude low‐coverage data, we set a commonly used threshold of at least 10 methylation sites per k‐mer per sample (Perez et al., 2023).

For our analysis of average DNA methylation across all annotated genes, we located the methylation context within a window of 4 kb upstream of the transcription start site (TSS) and a window of 4 kb downstream of the transcription termination site (TTS). Then, we looped over all genes to compute average methylation proportions on genomic windows (4 kb) upstream, downstream and inside of genes. We computed the average CG gene methylation across all genes and performed a binomial test (Takuno & Gaut, 2012) to assess whether coding regions had a significantly higher proportion of methylated cytosines than the genome‐wide background level of non‐coding regions. As methylation levels differ by developmental stage and caste, we performed binomial tests separately by stages and queens (Bewick et al., 2016; Muyle et al., 2021). This was performed for each cytosine in a CpG context and *p*‐values were corrected for multiple testing using Benjamini and Hochberg (1995) correction for each stage separately. Then we classified gene methylation into two categories: (1) Gene Body Methylated (GBM), if the adjusted *p*‐value for CpG methylation was higher than expected by chance, and (2) Un‐methylated (UM), if the adjusted *p*‐value for CG methylation was lower than expected by chance.

Finally, a GO term enrichment analysis was performed for all genes with significant gene‐body methylation, using the *topGO* package (2.56) (Alexa & Rahnenfuhrer, 2024), in R (4.5.0). Tests for overrepresented GO terms in Cellular Component, Biological Process, and Molecular Function were calculated using Fisher's exact test with hierarchical correction (weight 01), which generates a table of the top 200 most enriched GO terms sorted by significance *p*‐value <0.05.

### DNMT1 and DNMT3 methylation frequency along life stages and castes

We calculated methylation frequency along life stages and castes of the maintenance (DNMT1) and de novo (DNMT3) DNA methyltransferases using the data generated with ONT.

### Inferences of gene promoter methylation

To study the methylation of promoters, we used a window of 200 bp upstream of the TSS. We computed an average proportion of methylated promoter CpG sites and compared the resulting values to the genome average using a similar strategy to the gene body methylation (GBM) described above (Bewick et al., 2016; Muyle et al., 2021). First, we used a binomial test (Takuno & Gaut, 2012) and corrected obtained *p*‐values for multiple tests using Benjamini and Hochberg (1995) (see section above for more details). Then, we classified gene promoter methylation into two categories: (1) Promoter Gene Methylated (PGM) if adjusted *p*‐values were higher than expected, and (2) Promoter Un‐methylated (PUM) if adjusted *p*‐values were lower than expected. We did this analysis for each gene promoter region of each life stage. A promoter was considered methylated if the value was above 3% for all the stages and castes. Similarly, a GO enrichment analysis was performed for all methylated promoters.

### Gene expression analysis

We used a *P. californicus* transcriptome generated from 22 founding queens of *P. californicus* collected in 2018 and 2019 at Pine Valley (32.822 N, −116.528 W; pleometrotic population, P‐population) and Lake Henshaw resort (33.232 N, −116.760 W; haplometrotic population, H‐population), California, USA. Raw RNA‐sequence reads were produced using the same process as Helmkampf et al. (2016). The reads were trimmed to exclude low‐quality reads using Trimmomatic (v.0.39) (Bolger et al., 2014) (parameters: leading = 10, trailing = 10, minlen = 50). The software HISAT2 (v.2.2.1) (Kim et al., 2015) was used to map the reads to the *P. californicus* reference genome (Erbii et al. unpublished). FeatureCounts (v.2.0.1) (Liao et al., 2014) was used to calculate raw counts per gene. Then we converted the raw counts to FPKM (Fragments Per Kilobase Million) for our gene expression data, calculating the length of each gene in base pairs and converting the gene length from base pairs to kilobases (kb) by dividing it by 1000. Furthermore, we calculated the total mapped reads by summing up raw counts of all genes to get the total number of reads mapped for each sample (total count across all genes). We then applied the FPKM formula as:

$$\text{FPKM}=\mathrm{Raw}\;\text{Counts}\times 10^{9}/\text{total mapped reads}\times \text{gene length in}\;\mathrm{kb}.$$

## RESULTS

The methylome of *P. californicus* was successfully characterized using a combination of long (ONT) and short reads (WGBS). Larvae, pupae, workers and individual queens were analysed using ONT sequencing. Additionally, we analysed the same two pupae pools both by ONT sequencing and by WGBS. Therefore, we conducted a direct correlation of ONT sequencing and WGBS, comparing overlap and efficiency of both techniques (Table 1). As a backbone for all analyses, we used a recently updated chromosome‐level genome assembly and its annotation for *P. californicus* (Pcal3.1, Errbii et al., 2021).

**FIGURE 1:**
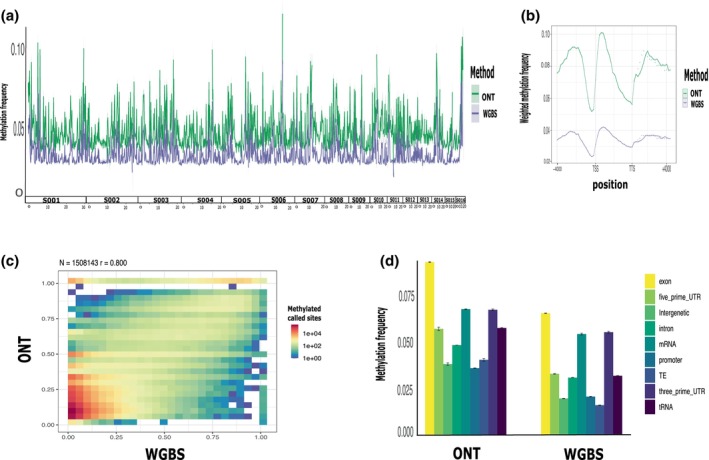
Methylome of *Pogonomyrmex californicus* with two sequencing methods Nanopore (ONT) and whole genome bisulfite sequencing (WGBS) using same DNA pupae pools (*n* = 2) comparing the efficiency of both methods to detect CpG methylation rates. (a) Average methylation frequency (ONT and WGBS) of two pupae pools along the genome (16 chromosome) (b) Weighted frequency methylation average (*n* = 2 pupae pools) (ONT and WGBS) looped over all genes region within a window of 4 kb upstream of the transcription start site (TSS) and a window of 4 kb downstream of the transcription end site (TTS) (c) Pearson correlation of methylation rates of the per‐CpG motif detected by both methods (ONT and WGBS), we discard the motif that were only detect by one method to do the correlation analysis (d) Average methylation frequency (*n* = 2 pupae pools) (ONT and WGBS) mapped by the genome region annotation.

WGBS is currently considered the gold standard to determine DNA methylation. We found that around 3% of cytosines in a CpG context were methylated using both methods (ONT and WGBS). However, WGBS found consistently fewer methylated sites in all genomic contexts (Figure 1a,b‐gene body,d‐intergenic sites), which could be explained in part by the lower coverage of WGBS (ONT sequencing: >40× coverage; 99% CpGs sites called after filtering and WGBS >20× coverage; 60% CpGs called after filtering) or due to inherent problems with the bisulfite treatment, which would corroborate another recent vertebrate study (López‐Catalina et al., 2024). Overall, CpG motif methylation sites detected by both methods in the same DNA pool were significantly correlated (Pearson correlation: *r* = 0.80, *p*‐value < 0.001, Figure 1a–c). Given that we analysed methylation calls derived from the same DNA pupal pool from *P. californicus* for both ONT sequencing and WGBS, our results demonstrate that ONT technology is a powerful, reliable and maybe even more sensitive tool to characterize insect methylomes.

On average, 18.3 million reads per adult sample were generated with ONT sequencing to detect 5mCpG (Table 1). The DNA methylation profile of *P. californicus* was characterized by genome‐wide low levels of CpG methylation (1%–10%), similar to what was observed in other hymenopteran species (Lewis et al., 2020). On average, 3% of all CpG sites were methylated (Table 1), with the highest methylation levels observed in genic regions (9.5%), followed by promoter regions (2.7%), and intergenic regions, with TEs being least methylated (2.5%) (Figure 1d). The gene bodies of the de novo DNA methyltransferase DNMT3 exhibited higher methylation frequencies across all developmental stages within the same population, as well as in queens from a different population, compared to the maintenance methyltransferases DNMT1 (Figure S2). These results sustained DNMT3 activity throughout development, which corroborates with a recent study where microRNA suppresses the DNA methyltransferase gene DNMT3, promoting queen‐like development in honeybee larvae (Jiang et al., 2025).

**FIGURE 2:**
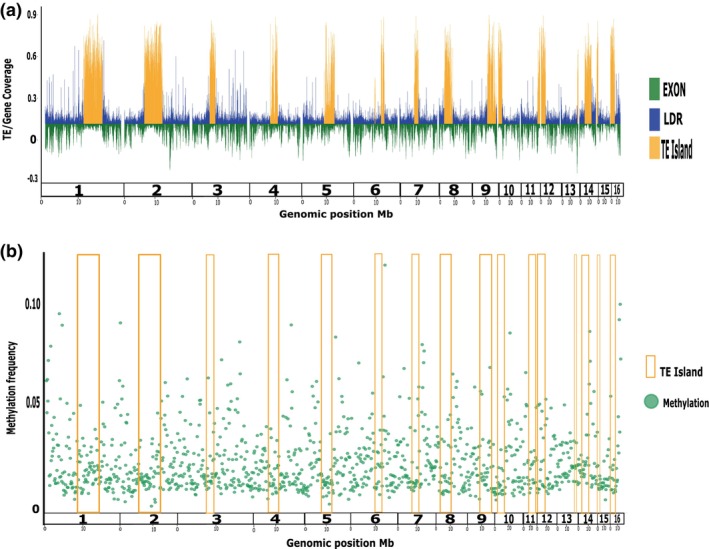
Transposable elements (TE) annotation and inference of TE islands along *Pogonomyrmex californicus* genome. (a) Mirror plot of the genomic positions (Mb) from long repeat regions (LDR, blue color) and TE islands regions (TE, yellow color) (above cero line), and the genome coverage (exons, green color [below cero line] along the genome. (b) Methylation frequency of CpG methylated sites (green dots) along the genomic regions, yellow squares represent the TE islands regions.

Methylation within genes was higher in exons than in introns, with the second and third exon showing the highest levels of methylation (10%) (Figure 1b,c, Figures S3 and S4). TEs, on the other hand, show low levels of methylation and a low methylation frequency in TE islands (2.5%) (Figures 1d and 2), but this was not significantly different when compared with the methylation frequency of the rest of the genome (W: 395784906, *p*‐value: 0.082). Such non‐significant differences could be explained by other genomic regions such as promoters or intergenic regions, which also have low average methylation frequencies. In addition, TE families differed in methylation frequencies. The highest methylation levels were found for Class I DNA transposons and the lowest methylation levels occurred in Class II retrotransposons (LTRs), but none was significantly different from the intergenic DNA methylation frequency (Figure S5). In addition, we found that TEs' average methylation in introns is lower than methylation on regions closer to the genes (2 kb upstream) and intergenetic regions; this is the case for all the stages, although these differences were not statistically significant (W: 290883410, *p* > 0.05) (Figure S6). TEs' average methylation along the genome did not differ significantly between stages (W: 450984515, *p* > 0.05) (Table S1).

**FIGURE 3:**
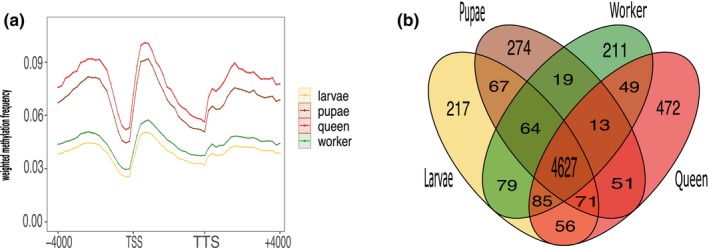
Gene methylation patterns along castes and life stages obtain with the sequencing method Oxford Nanopore Technologies (ONT). (a) Weighted frequency methylation average among life stages and castes (*n* = 2 pupae pools, 2 larva pools, 2 worker pools, 10 individuals queens) looped over all genes region within a window of 4 kb upstream of the transcription start site (TSS) and a window of 4 kb downstream of the transcription end site (TTS). (b) Venn diagram of genes body methylated unique and share along stages and castes (larva = yellow, pupae = brown, workers = green, queen = red).

Genome‐wide methylation levels varied among developmental stages and reproductive castes, with larvae showing the lowest percentage of methylated CpG sites (2.9%), followed by workers (3.1%), pupae (3.3%), and highest in queens (3.4%). However, the overall number of methylated and unmethylated genes did not differ between samples, despite the queens originating from a different population than the other developmental stages (Table 1). Weighted (average) methylation for gene regions was highest for queens and pupae and lowest for workers and larvae (Figure 3a).

To investigate gene methylation patterns across different life stages and castes, we first computed the average CpG DNA methylation across all genes and performed a binomial test to assess whether coding regions had a significantly higher proportion of methylated cytosines than the genome‐wide background level of non‐coding regions, which we then called methylated genes (GBM, gene body methylated) in contrast to other genes, which were lumped into the UM category (Bewick et al., 2016; Muyle et al., 2021) (see methods above for details). These analyses revealed 5024 methylated genes versus 10,875 unmethylated genes for larvae (Figure 3b, Table 1, Figure S7), 5186 GBM versus 10,713 UM genes (67%) for workers, 5320 GBM versus 9493 UM, and 5260 GBM versus 10,639 UM for queens (Figure 3b, Table 1, Figure S7). Interestingly, 91% (4698) of the GBM genes were shared between all stages. These shared genes were enriched for functional categories associated with housekeeping processes, including DNA replication, translation and transcription factors, RNA processing, and genes corresponding to methyltransferases (Appendix 1: GBM_commongenes_GO.terms.enrichment). It is important to acknowledge that some of the differences in gene methylation observed between developmental stages (larvae, pupae, workers) and queens may be influenced by population‐level variation. Previous studies have shown that up to 17% of DNA methylation can differ between colonies (Duncan et al., 2022). Nonetheless, it is noteworthy that queens—despite originating from a different population—shared most of their methylated genes with the other developmental stages. Queens also exhibited unique sets of genes (Figure 3b, Table 1, Figure S7), highlighting both commonality and stage‐specific epigenetic signatures.

We found unique GBM gene enrichments for each developmental stage (Figure 3b). In larvae, unique GBM genes were associated with lipid metabolism, RNA and DNA synthesis (housekeeping), and glycoproteins (immune system) involved in cell growth (Figure 3b, Table S2, Figure S8). In pupae, unique GBM genes were associated with glycosylase activity, metallopeptidase activity, ATP transporters, cell cycle, and proteolysis (Figure 3b, Table S3, Figure S9). Despite originating from different populations, workers and queens each shared unique GBM‐associated genes, which were enriched for functions related to olfactory receptor activity and biosynthetic processes (Figure 3b, Tables S4 and S5, Figures S10 and S11).

Promoter regions, defined as a region 200 bp upstream of the TSS, which showed a notable dip in DNA methylation, showed the lowest average methylation across all stages. In larvae, we found, analogous to GBM, 1007 Promoter Methylated Genes (PMG) (6.4%) and 14,712 Unmethylated Promoter Genes (PUM) (93,6%), while in pupae, there were 1102 PMG (7%) and 14,797 PUM (93%), and in workers, we identified 1492 PMG (9%) and 14,407 PUM (91%) (Figures S12 and S13). We also identified PGM genes unique to each stage. In larva, PMG genes were linked to transcription factors, DNA repair, glucose metabolism, and the Krebs cycle (Figure S14). In pupae, PMG genes were associated with DNA replication, transcription, and transmembrane transporters (Figure S15). Finally, in adults (workers), PMG genes were enriched in functions related to sensory perception of smell, chemoreception, gustatory receptors and odorant binding (Figure S16).

**FIGURE 4:**
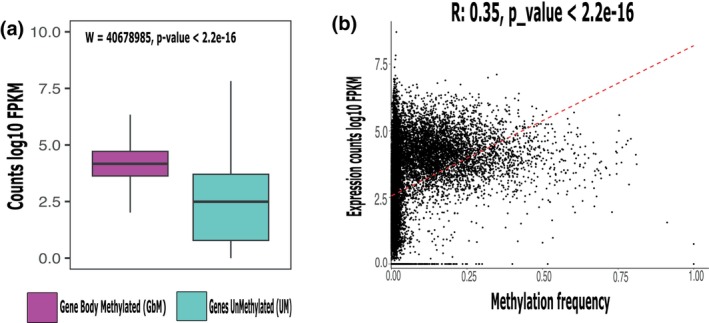
Gene expression level in *Pogonomyrmex californicus* queens generated 8–12 million RNA sequencing reads per sample (*n* = 22). (a) Expression levels in fragments per kilobase Million (FPKM) were higher in gene body methylated (GBM, pink) compared to unmethylated genes (UM, turquoise). (b) Pearson correlation was significantly positive between the expression level (FPKM) and methylation frequency of genes.

To study the effect of DNA methylation on gene expression in *P. californicus*, we generated 8–12 million RNA sequencing reads per founding queen (*n* = 22), with an average of 8.5 million reads per sample. After removing low‐quality reads, we obtained read counts for 15,948 transcripts. We found that GBM genes show a higher expression level (FPKM) compared to UM genes (W: 29872456 *p*‐value: <2.26e‐10; Figure 4a), consistent with the role of GBM in the upregulation of gene expression in other insects (Bonasio et al., 2012; Lyko et al., 2010; Suzuki & Bird, 2008; Zemach et al., 2010). Additionally, we found a positive correlation between the expression level and methylation frequency of genes (Pearson‐Correlation: *r* = 0.35 *p‐*value <2.2e‐16, Figure 4b). Although not significant (W = 6,193,912, *p* > 0.05), genes with their promoter region methylated show a slightly higher expression level (Figure S17).

## DISCUSSION

This study is the first to use ONT sequencing to detect DNA methylation in ants. We selected the California harvester ant, *P. californicus*, as it is a powerful model for investigating the epigenetic underpinnings of phenotypic plasticity in the context of social polymorphism. As noted above, populations of this species exhibit striking social polymorphism: colonies can be either haplometrotic, with a single reproductive queen, or pleometrotic, containing several unrelated queens (Overson et al., 2016). Because both social forms coexist sympatrically and hybridize (Errbi et al., 2024), *P. californicus* presents an ideal system for examining how DNA methylation may contribute to the developmental and behavioural plasticity associated with alternative social structures, which is an aspect of eusociality that remains largely unexplored at the epigenomic level. Furthermore, we found a highly significant correlation between ONT sequencing and the current gold standard in DNA methylation sequencing, WGBS (*r* = 0.8). This proves that ONT sequencing is well‐suited for DNA methylation studies in ants.

On average, 3% of the *P. californicus* genome‐wide CpG sites were methylated, which corroborates results of other Hymenopteran studies (Bewick et al., 2017). Gene regions, and in particular gene bodies, had the highest methylation frequency. Promoter regions, intergenic regions, and TEs have lower DNA methylation levels (Figures 1 and 2). In addition, we could show that GBM significantly increased gene expression levels in *P. californicus* queens (Figure 4), which has also been found in other insect species. Hence, it has been suggested that a reduced methylation frequency of TEs is an epigenetic way ‘to control and silence TE activity via RNA‐directed targeting mechanisms’ (Deniz et al., 2019; Perez et al., 2023). This is just the opposite way as DNA methylation functions in vertebrates, where an increase in DNA methylation reduces TE expression, but the concrete mechanism is unknown. We found that TEs exhibit higher average methylation levels in non‐TE intronic regions compared to intronic regions containing TEs (Figure S6), supporting the hypothesis that DNA methylation may play a role in silencing TEs. However, given that TE methylation levels vary considerably among invertebrate species and that some insects lack detectable DNA methylation altogether, the role of DNA methylation in TE regulation remains uncertain. Furthermore, detailed and tissue‐specific studies are needed to clarify the relationship between DNA methylation and TE expression in invertebrates (de Mendoza et al., 2020; Lewis et al., 2020; Lyko et al., 2010; Perez et al., 2023).

Developmental stages show distinct methylation frequencies, which may hint at an important intermediary role of DNA methylation for caste‐specific development and its function for caste determination in social insects (Kozeretska et al., 2017; Morandin et al., 2019; Yan et al., 2015). In our study, DNA methylation levels were lower in larvae. This pattern is consistent with observations in the juvenile stages of both vertebrates and invertebrates (Perez et al., 2023; Planques et al., 2021; Smallwood & Kelsey, 2012; Wang & Bhandari, 2019), where most DNA methylation marks are erased during gametogenesis and subsequently re‐established during development (Smallwood & Kelsey, 2012). However, this trend has not been clearly confirmed in insects. Nevertheless, it has been observed that juvenile stages—such as larvae and pupae—of social insects typically exhibit fewer morphological, physiological, and behavioural variations than adults, which may reduce the need for extensive gene expression (Harrison et al., 2015; Morandin et al., 2014). For example, Schrader et al. (2014) found that queens expressed significantly more genes than larvae in TE islands of the ant *Cardiocondyla obscurior*. Worker DNA methylation was lower than in queens and pupae, likely reflecting the reduced investment in growth and somatic maintenance typical of the worker caste (Harrison et al., 2015; Morandin et al., 2014), which diminishes the need for increased gene expression activity. In contrast, both pupae and queens are metabolically highly active, queens because they produce all eggs and pupae because they need to completely reorganize their anatomy and morphology.

Our study found that most genes with GBM (91%) were shared across all developmental stages and populations (queens were from a different population). These conserved GBM genes were significantly enriched for functional categories related to core cellular processes, including DNA replication, translation, transcription regulation, RNA processing and methyltransferase activity. These findings are consistent with previous studies in insects, which have shown that highly methylated genes tend to be evolutionarily conserved and broadly, stably expressed, rather than restricted to tissue‐specific or condition‐specific expression (Bonasio et al., 2012; Duncan et al., 2022; Glastad et al., 2014; Lewis et al., 2020; Lyko et al., 2010; Morandin et al., 2019).

Even so, we detected stage‐specific differences. Larvae show GBM enrichment in genes involved in lipid metabolism and storage, immune‐related glycoproteins, and phosphorylation synthesis that support rapid cell growth. In pupae, GBM is concentrated in genes linked to glycosylase activity and cell‐cycle progression, reflecting intense tissue remodelling. These pattern parallel findings from other ants and social insects, where caste‐ and stage‐specific expression of growth‐ and development‐related genes have been documented. Gstöttl et al. (2020) and Morandin et al. (2019) also found that larvae and pupae upregulated genes involved in growth and tissue buildup. Similarly, for several other social insect species, Kapheim (2017) documented that larvae have upregulated genes related to lipid storage and lipid metabolism.

Despite originating from different populations, workers and queens shared unique GBM‐associated genes that were enriched in functional categories related to chemical communication, including chemoreception, gustatory receptors and odorant‐binding proteins. These categories were also enriched in a transcriptomic study of *P. californicus* founding queens (Helmkampf et al., 2016). Chemical communication has several specific functions in social insects, example., nestmate discrimination or caste recognition. Furthermore, queens were observed to have uniquely methylated genes involved in metabolic processes, DNA damage checkpoints, and metalloproteases, which are involved in the modulation of cell growth, inflammation, immunity and hormone processing. This coincides with transcriptomic studies that have found that queens upregulated DNA repair genes (Gstöttl et al., 2020), which have been associated with anti‐ageing strategies (Gstöttl et al., 2020; Lucas et al., 2016).

Body methylated genes, shared across all developmental stages, were enriched for functional categories related to core housekeeping processes, including DNA and RNA processing, cell cycle regulation, mitochondrial respiration, HsP30 genes and several methyltransferases (Appendix 1: GBM_commongenes_GO.terms.enrichment). This aligns with previous studies, which have shown that genes involved in housekeeping functions tend to exhibit the highest levels of GBM (Perez et al., 2023). Furthermore, upregulation of genes linked to ‘Regulation of Gene Expression and Epigenetic Mechanisms’, has been related to a high fecundity and a long life in social insects (Gstöttl et al., 2020).

The methylation of the promoter region has also been associated with an increase in gene expression in insects (Bewick et al., 2017). In the harvester ant *P. californicus*, we found a positive trend—but no significant relationship—between promoter methylation and higher expression. Some studies have suggested that promoter methylation plays a supporting role for other gene expression mechanisms, as these also found no evidence of promoter methylation and gene expression or silencing (Keller et al., 2016; Perez et al., 2023).

Both *DNMT1* and *DNMT3* were methylated in *P. californicus*, with *DNMT3* showing a markedly higher methylation frequency (Figure S2). Interestingly, both genes have significantly diverged between populations and social forms, accumulating non‐synonymous substitutions that may reflect functional differentiation (Errbi et al., 2024). Genetic variation can influence DNA methylation and gene expression, potentially linking genotypic differences to social polymorphism through epigenetic mechanisms (Wedd et al., 2016). The maintenance methyltransferase *DNMT1* preserves methylation patterns during insect development and regulates genes involved in the division of labour in honeybees (Lyko et al., 2010). However, knockout studies suggest that gene‐body methylation is not essential for normal somatic gene expression (Bewick et al., 2019).

In Hymenoptera, *DNMT3* has evolved lineage‐specific innovations suggesting that DNMTs may have acquired wider regulatory functions related to chromatin dynamics, transcriptional control or developmental signalling (Del Castillo Falconi et al., 2022; Duymich et al., 2016; Kucharski et al., 2023). However, how these molecular innovations interact with other epigenetic mechanisms remains unclear. For instance, a recent study in *A. mellifera* showed that a microRNA derived from TEs can suppress *DNMT3*, influencing queen‐like development (Jiang et al., 2025).

Beyond DNMTs, Romiguier et al. (2022) identified positive gene selection on numerous histone‐modifying enzymes throughout ant evolution, suggesting adaptive changes in chromatin regulatory pathways associated with eusociality. Remarkably, similar genes were also gene‐body methylated in *P. californicus* (Appendix 1: GBM_commongenes_GO.terms.enrichment), pointing to complex, interconnected epigenetic networks. Despite growing evidence, the interactions among these pathways—and their collective role in shaping caste differentiation and social complexity—remain poorly understood. Unravelling these relationships will be essential for understanding how epigenetic mechanisms contribute to the evolution of eusociality and complex social behaviours in ants.

## CONCLUSION

The harvester ant *P. californicus*, like other solitary and social Hymenoptera (bumble bees, honeybees and the parasitoid wasp *Nasonia*), has a small but significant proportion of its CpGs methylated. DNA methylation is, like in other invertebrates, highly concentrated in exons, and gene expression in *P. californicus* increases with GBM. We could show that DNA methylation varies between developmental stages and is lowest in larvae, followed by adult workers, pupae and queens. As DNA methylation is known to correlate with population‐level genetic variation, we cannot definitively attribute the differences in DNA methylation between workers and queens to caste‐specific patterns. Nonetheless, despite their distinct geographic origins, workers and queens shared a large proportion of overlapping sets of methylated genes, particularly those involved in olfactory receptor activity and biosynthetic processes. These functional categories were also enriched in a transcriptomic study of *P. californicus* founding queens (Helmkampf et al., 2016), suggesting that DNA methylation may play a meaningful role in caste differentiation, even if population effects cannot be entirely ruled out.

These findings underscore the need for further research into how DNA methylation integrates within broader epigenetic networks and how these processes influence gene expression and phenotypic plasticity in ants, particularly in *P. californicus*. Owing to its remarkable behavioural and social variation—including intraspecific social polymorphism—*P. californicus* represents an exceptional model for investigating the epigenetic basis of phenotypic diversity. Continued studies on this species will advance our understanding of how epigenetic modifications shape development, caste differentiation and the evolution of complex social organization in eusocial insects.

## AUTHOR CONTRIBUTIONS

**Tania Chavarria‐Pizarro:** Writing – original draft; writing – review and editing; investigation; methodology; visualization; formal analysis. **Mohammed Errbii:** Methodology; writing – review and editing; data curation; formal analysis. **Janina Rinke:** Formal analysis; methodology; writing – review and editing; visualization. **Lukas Schrader:** Writing – review and editing; formal analysis. **Jürgen Gadau:** Conceptualization; funding acquisition; writing – review and editing; resources; supervision; project administration.

## CONFLICT OF INTEREST STATEMENT

The authors declare no conflicts of interest.

## Supporting information


**Figure S1.** Relative content of transposable elements (TEs pink) and genes (turquoise) along 10 scaffolds (chromosomes) using a 50 kb long genomic window along the genome of *P. californicus*.
**Figure S2**. DNMT1 and DNMT3 methylation frequency in *P. californicus* and phylogenetic similarity with close related species. (A) Average methylation frequency (%) of DNMT1 and DNMT3 along life stages (*n* = 2 pupae pools, 2 larva pools, adults = 2 worker pools + 10 individuals queens). (B) DNMT1 maximum likelihood phylogenetic tree along some related Hymenopteran species with DNMT1 sequences available. (C) DNMT3b maximum likelihood phylogenetic tree along some related Hymenopteran species with DNMT3b sequences available.
**Figure S3**. Average methylation frequency of exons and introns along *P. californicus* genome. (A) Methylation frequency of exons and intros according with the CpG Density, on the *x*‐axis we plot the methylation frequency calculated with ONT and on the *y*‐axis we plotted the density (CpG methylated sites/total CpG sites) on percentages (Exons = dark green, Introns = light green). (B) methylation frequency of exons and introns along the 16 chromosomes.
**Figure S4**. Average methylation frequency and standard deviation by number of exons and introns.
**Figure S5**. Average methylation frequency and standard deviation of transposable elements (TEs) families in *P. californicus* genomes.
**Figure S6**. Average methylation frequency and standard deviation of transposable elements (TEs) in *P. californicus* genome regions (Intron, Intergenetic, and a window 2 kb upstream the gene region).
**Figure S7**. Gene methylation patterns along life stages obtain with the sequencing method ONT. (A) Venn diagram of Genes body methylated unique and share along stages and castes (larva = yellow, pupae = brown, adults = red) (*n* = 2 pupae pools, 2 larva pools, adults = 2 worker pools). (B) Venn diagram of genes body unmethylated unique and share along stages and castes (larva = yellow, pupae = brown, adults = red) (*n* = 2 pupae pools, 2 larva pools, adults = 2 worker pools).
**Figure S8**. GO enrichment analysis for all genes body methylated unique to larvae (*n* = 2 larvae pools) with significant gene‐body methylation with a *p*‐value <0.05 representing significantly enriched GO terms.
**Figure S9**. GO enrichment analysis for all genes body methylated unique to pupae (*n* = 2 pupae pooles) with significant gene‐body methylation with a *p*‐value <0.05 representing significantly enriched GO terms.
**Figure S10**. GO enrichment analysis for all genes body methylated unique to queen (*n* = 10 individual queens) with significant gene‐body methylation with a *p*‐value <0.05 representing significantly enriched GO terms.
**Figure S11**. GO enrichment analysis for all genes body methylated unique to workers (*n* = 2 worker pools) with significant gene‐body methylation with a *p*‐value <0.05 representing significantly enriched GO terms.
**Figure S12**. Promoter methylated genes patterns along life stages obtain with the sequencing method ONT. (A) Venn diagram of Promoter methylated genes unique and share along stages and castes (larva = yellow, pupae = brown, adults = red) (*n* = 2 pupae pools, 2 larva pools, adults = 2 worker pools). (B) Venn diagram of Promoter Unmethylated genes unique and share along stages and castes (larva = yellow, pupae = brown, adults = red) (*n* = 2 pupae pools, 2 larva pools, adults = 2 worker pools).
**Figure S13**. GO enrichment analysis for all genes with significant promoter methylation common to all life stages (*n* = 2 pupae pools, 2 larva pools, adults = 2 worker pools) *p*‐value <0.05 representing significantly enriched GO terms.
**Figure S14**. GO enrichment analysis for all genes unique to larvae with significant promoter methylation (*n* = 2 larvae pools) *p*‐value <0.05 representing significantly enriched GO terms.
**Figure S15**. GO enrichment analysis for all genes unique to pupae with significant promoter methylation (*n* = 2 pupae pools) *p*‐value <0.05 representing significantly enriched GO terms.
**Figure S16**. GO enrichment analysis for all genes unique to workers with significant promoter methylation (adults = 2 worker pools) *p*‐value <0.05 representing significantly enriched GO terms.
**Figure S17**. Gene expression level of genes with promoter methylation in *P. californicus* queens generated 8‐12 million RNA sequencing reads per sample (*n* = 22). Expression levels in fragments per kilobase Million (FPKM) were non‐significant higher in gene with promoter methylation (pink) compared to Unmethylated promoter genes (turquoise).
**Table S1**. Average methylation and standard error of transposable elements along three regions (Intron, inter‐genetic, and a window of 2 kb upstream of the gene region between stages).
**Table S2**. Gene differential methylated unique for larvae significant associated to ontology using a Fisher test. GO.ID (GO Term Identification number), GOTerm, NumDMGs (number differential body methylated genes), pvalue (Fisher test).
**Table S3**. Gene differential methylated unique for pupae significant associated to ontology using a Fisher test. GO.ID (GO Term Identification number), GOTerm, NumDMGs (number methylated genes), pvalue (Fisher test).
**Table S4**. Gene differential methylated unique for worker significant associated to ontology using a Fisher test. GO.ID (GO Term Identification number), GO Term, NumDMGs (number methylated genes), pvalue (Fisher test).
**Table S5**. Gene differential methylated unique for queen significant associated to ontology using a Fisher test. GO.ID (GO Term Identification number), GOTerm, NumDMGs (number methylated genes), pvalue (Fisher test).

**Appendix 1.** Gene differential methylated share for all developmental stages significant associated to Ontology using a Fisher test. GO.ID (GO Term Identification number), GoTerm (category), GOTerm, NumDMGs (number differential body methylated genes), pvalue (Fisher test), Ontology.

## Data Availability

The required scripts to do the methylation analysis (e.g., basecalling, methylation calling, bedtools, samtools and R script) are available in this repository https://github.com/TaniaChP79/P.cal-methylome (Chavarria‐Pizarro et al., 2025a) The transcriptome data and methylation calls have been deposited with link https://doi.org/10.6084/m9.figshare.29820020 (Chavarria‐Pizarro et al., 2025b) and they are available for release now.
