# Supplementary material for: Genome‐wide and gene‐specific DNA methylation across developmental stages in Pogonomyrmex californicus: A socially polymorphic ant
Source: Insect Mol Biol. 2025 Dec 20;35(3):232–45. doi: 10.1111/imb.70021 (PMC13139995; doi:10.1111/imb.70021)
Supplement: Supplementary file 2 — Appendix 1. Gene differential methylated share for all developmental stages significant associated to Ontology using a Fisher test. GO.ID (GO Term Identification number), GoTerm (category), GOTerm, NumDMGs (number differential body methylated genes), pvalue (Fisher test), Ontology. [file IMB-35-232-s001.pdf]

Appendix 1. Gene differential methylated share for all developmental stages significant associated to Ontology using a Fisher test. GO.ID (GO Term Identification number), GoTerm(category),GOTerm, NumDMGs (number differential body methylated genes), pvalue (Fisher test), Ontology.

GO.ID	GOTerm	NumDMGs	pvalue	Ontology
GO:0003723	RNA binding	354	9,3e-27	Molecular Function
GO:0005515	protein binding	1750	2,4e-12	Molecular Function
GO:0003724	RNA helicase activity	37	2,4e-10	Molecular Function
GO:0005524	ATP binding	664	4,3e-10	Molecular Function
GO:0061630	ubiquitin protein ligase activity	39	6e-09	Molecular Function
GO:0005525	GTP binding	152	3e-08	Molecular Function
GO:0016887	ATP hydrolysis activity	99	5,7e-08	Molecular Function
GO:0051082	unfolded protein binding	25	8,9e-07	Molecular Function
GO:0003924	GTPase activity	124	2,5e-06	Molecular Function
GO:0004843	cysteine-type deubiquitinase activity	34	1,6e-05	Molecular Function
GO:0051539	4 iron, 4 sulfur cluster binding	12	1,6e-05	Molecular Function
GO:0140662	ATP-dependent protein folding chaperone	21	2,1e-05	Molecular Function
GO:0003684	damaged DNA binding	11	4,1e-05	Molecular Function
GO:0003743	translation initiation factor activity	29	9e-05	Molecular Function
GO:0000287	magnesium ion binding	30	0,00021	Molecular Function
GO:0003899	DNA-directed 5'-3' RNA polymerase activi,,,	16	0,00031	Molecular Function
GO:0035091	phosphatidylinositol binding	37	0,00039	Molecular Function
GO:0003887	DNA-directed DNA polymerase activity	8	0,00064	Molecular Function
GO:0003712	transcription coregulator activity	49	0,00113	Molecular Function
GO:0003697	single-stranded DNA binding	14	0,00128	Molecular Function
GO:0003755	peptidyl-prolyl cis-trans isomerase acti,,,	27	0,00132	Molecular Function
GO:0016779	nucleotidyltransferase activity	47	0,00155	Molecular Function

GO:0000049	tRNA binding	10	0,00165	Molecular Function
GO:0019843	rRNA binding	16	0,00273	Molecular Function
GO:0005484	SNAP receptor activity	9	0,00374	Molecular Function
GO:0003746	translation elongation factor activity	9	0,00374	Molecular Function
GO:0031625	ubiquitin protein ligase binding	9	0,00374	Molecular Function
GO:0043130	ubiquitin binding	14	0,00382	Molecular Function
GO:0016409	palmitoyltransferase activity	14	0,00382	Molecular Function
GO:0004518	nuclease activity	55	0,00389	Molecular Function
GO:0015078	proton transmembrane transporter activit,,,	34	0,00397	Molecular Function
GO:0003690	double-stranded DNA binding	26	0,00403	Molecular Function
GO:0002161	aminoacyl-tRNA editing activity	7	0,00404	Molecular Function
GO:0036402	proteasome-activating activity	6	0,00405	Molecular Function
GO:0008312	7S RNA binding	6	0,00405	Molecular Function
GO:0046961	proton-transporting ATPase activity, rot,,,	16	0,00478	Molecular Function
GO:0017056	structural constituent of nuclear pore	11	0,00582	Molecular Function
GO:0008408	3'-5' exonuclease activity	15	0,00833	Molecular Function
GO:0043022	ribosome binding	10	0,00838	Molecular Function
GO:0016274	protein-arginine N-methyltransferase act,,,	8	0,00841	Molecular Function
GO:0004176	ATP-dependent peptidase activity	5	0,01014	Molecular Function
GO:0046933	proton-transporting ATP synthase activit,,,	5	0,01014	Molecular Function
GO:0005049	nuclear export signal receptor activity	5	0,01014	Molecular Function
GO:0004842	ubiquitin-protein transferase activity	72	0,01194	Molecular Function
GO:0003678	DNA helicase activity	14	0,01208	Molecular Function
GO:0009982	pseudouridine synthase activity	10	0,01212	Molecular Function
GO:0005096	GTPase activator activity	34	0,01986	Molecular Function
GO:0004519	endonuclease activity	26	0,02515	Molecular Function
GO:0004721	phosphoprotein phosphatase activity	53	0,02537	Molecular Function

GO:0030515	snoRNA binding	6	0,02537	Molecular Function
GO:0004525	ribonuclease III activity	4	0,02542	Molecular Function
GO:0004298	threonine-type endopeptidase activity	4	0,02542	Molecular Function
GO:0003756	protein disulfide isomerase activity	4	0,02542	Molecular Function
GO:0003725	double-stranded RNA binding	4	0,02542	Molecular Function
GO:0017025	TBP-class protein binding	4	0,02542	Molecular Function
GO:0004826	phenylalanine-tRNA ligase activity	4	0,02542	Molecular Function
GO:0008137	NADH dehydrogenase (ubiquinone) activity	4	0,02542	Molecular Function
GO:0008168	methyltransferase activity	106	0,02596	Molecular Function
GO:0016787	hydrolase activity	1175	0,02765	Molecular Function
GO:0016538	cyclin-dependent protein serine/threonin,,,	13	0,0289	Molecular Function
GO:0016780	phosphotransferase activity, for other s,,,	13	0,04035	Molecular Function
GO:0000030	mannosyltransferase activity	14	0,04037	Molecular Function
GO:0051879	Hsp90 protein binding	6	0,04063	Molecular Function
GO:0000166	nucleotide binding	945	0,04083	Molecular Function
GO:0030234	enzyme regulator activity	169	0,04874	Molecular Function
GO:0009055	electron transfer activity	15	0,04907	Molecular Function
GO:0051087	protein-folding chaperone binding	13	0,04926	Molecular Function
GO:0006412	translation	221	1,1e-25	Biological Process
GO:0006364	rRNA processing	46	1e-10	Biological Process
GO:0006886	intracellular protein transport	83	1,8e-09	Biological Process
GO:0006457	protein folding	50	1,5e-08	Biological Process
GO:0006260	DNA replication	58	1,8e-07	Biological Process
GO:0006281	DNA repair	104	3,9e-07	Biological Process
GO:0006397	mRNA processing	105	7,5e-07	Biological Process
GO:0000398	mRNA splicing, via spliceosome	69	1e-06	Biological Process
GO:0006511	ubiquitin-dependent protein catabolic pr,,,	75	2,4e-06	Biological Process

GO:0006888	endoplasmic reticulum to Golgi vesicle-m,,,	18	4,9e-06	Biological Process
GO:0006289	nucleotide-excision repair	13	2,5e-05	Biological Process
GO:0016192	vesicle-mediated transport	147	3e-05	Biological Process
GO:0006352	DNA-templated transcription initiation	34	5,3e-05	Biological Process
GO:0006351	DNA-templated transcription	437	0,00012	Biological Process
GO:0016579	protein deubiquitination	33	0,00013	Biological Process
GO:0016226	iron-sulfur cluster assembly	13	0,00013	Biological Process
GO:0015986	proton motive force-driven ATP synthesis	11	0,00013	Biological Process
GO:0016567	protein ubiquitination	45	0,00017	Biological Process
GO:0042254	ribosome biogenesis	69	0,00018	Biological Process
GO:0006367	transcription initiation at RNA polymera,,,	12	0,00029	Biological Process
GO:0043161	proteasome-mediated ubiquitin-dependent ,,,	22	0,00065	Biological Process
GO:0006396	RNA processing	269	0,00067	Biological Process
GO:0008033	tRNA processing	55	0,00084	Biological Process
GO:0006913	nucleocytoplasmic transport	43	0,00143	Biological Process
GO:0006614	SRP-dependent cotranslational protein ta,,,	9	0,00149	Biological Process
GO:0043248	proteasome assembly	9	0,00149	Biological Process
GO:0006606	protein import into nucleus	8	0,00149	Biological Process
GO:0002098	tRNA wobble uridine modification	12	0,00191	Biological Process
GO:0006506	GPI anchor biosynthetic process	24	0,00227	Biological Process
GO:0034314	Arp2/3 complex-mediated actin nucleation	8	0,00336	Biological Process
GO:0032508	DNA duplex unwinding	8	0,00336	Biological Process
GO:0006890	retrograde vesicle-mediated transport, G,,,	7	0,00336	Biological Process
GO:0015031	protein transport	108	0,00612	Biological Process
GO:0006383	transcription by RNA polymerase III	12	0,00752	Biological Process
GO:0032543	mitochondrial translation	6	0,0076	Biological Process
GO:0007034	vacuolar transport	14	0,00805	Biological Process

GO:0006414	translational elongation	13	0,00807	Biological Process
GO:0042147	retrograde transport, endosome to Golgi	9	0,00813	Biological Process
GO:0000184	nuclear-transcribed mRNA catabolic proce,,,	9	0,00813	Biological Process
GO:1902600	proton transmembrane transport	19	0,0093	Biological Process
GO:0000413	protein peptidyl-prolyl isomerization	16	0,01297	Biological Process
GO:0006270	DNA replication initiation	13	0,01314	Biological Process
GO:0001522	pseudouridine synthesis	12	0,01316	Biological Process
GO:0006486	protein glycosylation	45	0,01432	Biological Process
GO:0008380	RNA splicing	80	0,01601	Biological Process
GO:0006366	transcription by RNA polymerase II	114	0,01604	Biological Process
GO:0006368	transcription elongation by RNA polymera,,,	11	0,01645	Biological Process
GO:0000387	spliceosomal snRNP assembly	9	0,01646	Biological Process
GO:0048193	Golgi vesicle transport	37	0,01648	Biological Process
GO:0006384	transcription initiation at RNA polymera,,,	5	0,01714	Biological Process
GO:0045292	mRNA cis splicing, via spliceosome	5	0,01714	Biological Process
GO:0001510	RNA methylation	28	0,02409	Biological Process
GO:0006338	chromatin remodeling	31	0,02995	Biological Process
GO:0030163	protein catabolic process	97	0,03045	Biological Process
GO:0006401	RNA catabolic process	36	0,03207	Biological Process
GO:0030833	regulation of actin filament polymerizat,,,	13	0,03287	Biological Process
GO:0018216	peptidyl-arginine methylation	8	0,03293	Biological Process
GO:0000724	double-strand break repair via homologou,,,	7	0,03298	Biological Process
GO:0001682	tRNA 5'-leader removal	7	0,03298	Biological Process
GO:0030488	tRNA methylation	7	0,03298	Biological Process
GO:0071985	multivesicular body sorting pathway	7	0,03856	Biological Process
GO:0016180	snRNA processing	7	0,03856	Biological Process
GO:0006098	pentose-phosphate shunt	5	0,03864	Biological Process

GO:0007099	centriole replication	4	0,03868	Biological Process
GO:0018344	protein geranylgeranylation	4	0,03868	Biological Process
GO:0006432	phenylalanyl-tRNA aminoacylation	4	0,03868	Biological Process
GO:0000381	regulation of alternative mRNA splicing,,,,	4	0,03868	Biological Process
GO:0051225	spindle assembly	4	0,03868	Biological Process
GO:0006269	DNA replication, synthesis of RNA primer	4	0,03868	Biological Process
GO:0036297	interstrand cross-link repair	4	0,03868	Biological Process
GO:0006241	CTP biosynthetic process	4	0,03868	Biological Process
GO:0015937	coenzyme A biosynthetic process	4	0,03868	Biological Process
GO:0000290	deadenylation-dependent decapping of nuc,,,	4	0,03868	Biological Process
GO:0000245	spliceosomal complex assembly	7	0,0387	Biological Process
GO:0006413	translational initiation	21	0,04307	Biological Process
GO:0005840	ribosome	142	6,9e-18	Cellular Component
GO:0005634	nucleus	472	2e-11	Cellular Component
GO:0005739	mitochondrion	133	3,3e-06	Cellular Component
GO:0015935	small ribosomal subunit	18	5,1e-05	Cellular Component
GO:0005852	eukaryotic translation initiation factor,,,	13	5,1e-05	Cellular Component
GO:0005643	nuclear pore	13	0,00011	Cellular Component
GO:0016592	mediator complex	25	0,00014	Cellular Component
GO:0015934	large ribosomal subunit	18	0,00104	Cellular Component
GO:0005669	transcription factor TFIID complex	9	0,00108	Cellular Component
GO:0032040	small-subunit processome	9	0,00108	Cellular Component
GO:0005681	spliceosomal complex	15	0,00159	Cellular Component
GO:0005794	Golgi apparatus	48	0,00458	Cellular Component
GO:0019773	proteasome core complex, alpha-subunit c,,,	7	0,00493	Cellular Component
GO:0032039	integrator complex	7	0,00493	Cellular Component
GO:0005730	nucleolus	15	0,00613	Cellular Component

GO:0000139	Golgi membrane	10	0,00624	Cellular Component
GO:0005829	cytosol	15	0,01029	Cellular Component
GO:0005839	proteasome core complex	14	0,01032	Cellular Component
GO:0005885	Arp2/3 protein complex	6	0,01055	Cellular Component
GO:0033588	elongator holoenzyme complex	6	0,01055	Cellular Component
GO:0042555	MCM complex	6	0,01055	Cellular Component
GO:0016272	prefoldin complex	6	0,01055	Cellular Component
GO:0005783	endoplasmic reticulum	59	0,01156	Cellular Component
GO:0005761	mitochondrial ribosome	14	0,02228	Cellular Component
GO:0030014	CCR4-NOT complex	8	0,0224	Cellular Component
GO:0022625	cytosolic large ribosomal subunit	5	0,02256	Cellular Component
GO:0046540	U4/U6 x U5 tri-snRNP complex	5	0,02256	Cellular Component
GO:0035267	NuA4 histone acetyltransferase complex	5	0,02256	Cellular Component
GO:0000276	mitochondrial proton-transporting ATP sy,,,	5	0,02256	Cellular Component
GO:0008180	COP9 signalosome	8	0,02333	Cellular Component
GO:0000145	exocyst	8	0,02333	Cellular Component
GO:0000124	SAGA complex	7	0,04426	Cellular Component
GO:0005747	mitochondrial respiratory chain complex ,,,	7	0,04426	Cellular Component
GO:0048500	signal recognition particle	6	0,04802	Cellular Component
GO:0030008	TRAPP complex	5	0,04811	Cellular Component
GO:0033180	proton-transporting V-type ATPase, V1 do,,,	5	0,04811	Cellular Component
GO:0071203	WASH complex	4	0,0482	Cellular Component
GO:0005869	dynactin complex	4	0,0482	Cellular Component
GO:0000172	ribonuclease MRP complex	4	0,0482	Cellular Component
GO:0005666	RNA polymerase III complex	4	0,0482	Cellular Component
GO:0030127	COPII vesicle coat	4	0,0482	Cellular Component
GO:0030915	Smc5-Smc6 complex	4	0,0482	Cellular Component

GO:0005762	mitochondrial large ribosomal subunit	4	0,0482	Cellular Component
GO:0005787	signal peptidase complex	4	0,0482	Cellular Component
GO:0000439	transcription factor TFIIH core complex	4	0,0482	Cellular Component
GO:0045261	proton-transporting ATP synthase complex,,,	4	0,0482	Cellular Component
